# Machine Learning–Derived Prenatal Predictive Risk Model to Guide Intervention and Prevent the Progression of Gestational Diabetes Mellitus to Type 2 Diabetes: Prediction Model Development Study

**DOI:** 10.2196/32366

**Published:** 2022-07-05

**Authors:** Mukkesh Kumar, Li Ting Ang, Cindy Ho, Shu E Soh, Kok Hian Tan, Jerry Kok Yen Chan, Keith M Godfrey, Shiao-Yng Chan, Yap Seng Chong, Johan G Eriksson, Mengling Feng, Neerja Karnani

**Affiliations:** 1 Singapore Institute for Clinical Sciences Agency for Science Technology and Research Singapore Singapore; 2 Bioinformatics Institute Agency for Science Technology and Research Singapore Singapore; 3 Saw Swee Hock School of Public Health National University of Singapore National University Health System Singapore Singapore; 4 Department of Paediatrics Yong Loo Lin School of Medicine National University of Singapore Singapore Singapore; 5 Division of Obstetrics and Gynecology KK Women’s and Children’s Hospital Singapore Singapore; 6 Obstetrics and Gynecology Academic Clinical Programme Duke-NUS Graduate Medical School Singapore Singapore; 7 Department of Obstetrics and Gynaecology and Human Potential Translational Research Programme Yong Loo Lin School of Medicine National University of Singapore Singapore Singapore; 8 Department of Reproductive Medicine KK Women’s and Children’s Hospital Singapore Singapore; 9 Cancer and Stem Cell Biology Duke-NUS Medical School Singapore Singapore; 10 MRC Lifecourse Epidemiology Unit University of Southampton Southampton United Kingdom; 11 National Institute for Health and Care Research Southampton Biomedical Research Centre University Hospital Southampton NHS Foundation Trust Southampton United Kingdom; 12 Department of General Practice and Primary Health Care University of Helsinki Helsinki Finland; 13 Folkhälsan Research Center Helsinki Finland; 14 Institute of Data Science National University of Singapore Singapore Singapore; 15 Department of Biochemistry Yong Loo Lin School of Medicine National University of Singapore Singapore Singapore

**Keywords:** Asian populations, diabetes management, digital health, gestational diabetes mellitus, machine learning, prediction models, prenatal care, public health, risk factors, type 2 diabetes

## Abstract

**Background:**

The increasing prevalence of gestational diabetes mellitus (GDM) is concerning as women with GDM are at high risk of type 2 diabetes (T2D) later in life. The magnitude of this risk highlights the importance of early intervention to prevent the progression of GDM to T2D. Rates of postpartum screening are suboptimal, often as low as 13% in Asian countries. The lack of preventive care through structured postpartum screening in several health care systems and low public awareness are key barriers to postpartum diabetes screening.

**Objective:**

In this study, we developed a machine learning model for early prediction of postpartum T2D following routine antenatal GDM screening. The early prediction of postpartum T2D during prenatal care would enable the implementation of effective strategies for diabetes prevention interventions. To our best knowledge, this is the first study that uses machine learning for postpartum T2D risk assessment in antenatal populations of Asian origin.

**Methods:**

Prospective multiethnic data (Chinese, Malay, and Indian ethnicities) from 561 pregnancies in Singapore’s most deeply phenotyped mother-offspring cohort study—Growing Up in Singapore Towards healthy Outcomes—were used for predictive modeling. The feature variables included were demographics, medical or obstetric history, physical measures, lifestyle information, and GDM diagnosis. Shapley values were combined with CatBoost tree ensembles to perform feature selection. Our game theoretical approach for predictive analytics enables population subtyping and pattern discovery for data-driven precision care. The predictive models were trained using 4 machine learning algorithms: logistic regression, support vector machine, CatBoost gradient boosting, and artificial neural network. We used 5-fold stratified cross-validation to preserve the same proportion of T2D cases in each fold. Grid search pipelines were built to evaluate the best performing hyperparameters.

**Results:**

A high performance prediction model for postpartum T2D comprising of 2 midgestation features—midpregnancy BMI after gestational weight gain and diagnosis of GDM—was developed (BMI_GDM CatBoost model: AUC=0.86, 95% CI 0.72-0.99). Prepregnancy BMI alone was inadequate in predicting postpartum T2D risk (ppBMI CatBoost model: AUC=0.62, 95% CI 0.39-0.86). A 2-hour postprandial glucose test (BMI_2hour CatBoost model: AUC=0.86, 95% CI 0.76-0.96) showed a stronger postpartum T2D risk prediction effect compared to fasting glucose test (BMI_Fasting CatBoost model: AUC=0.76, 95% CI 0.61-0.91). The BMI_GDM model was also robust when using a modified 2-point International Association of the Diabetes and Pregnancy Study Groups (IADPSG) 2018 criteria for GDM diagnosis (BMI_GDM2 CatBoost model: AUC=0.84, 95% CI 0.72-0.97). Total gestational weight gain was inversely associated with postpartum T2D outcome, independent of prepregnancy BMI and diagnosis of GDM (P=.02; OR 0.88, 95% CI 0.79-0.98).

**Conclusions:**

Midgestation weight gain effects, combined with the metabolic derangements underlying GDM during pregnancy, signal future T2D risk in Singaporean women. Further studies will be required to examine the influence of metabolic adaptations in pregnancy on postpartum maternal metabolic health outcomes. The state-of-the-art machine learning model can be leveraged as a rapid risk stratification tool during prenatal care.

**Trial Registration:**

ClinicalTrials.gov NCT01174875; https://clinicaltrials.gov/ct2/show/NCT01174875

## Introduction

The prevalence of gestational diabetes mellitus (GDM) is increasing globally, with 1 in 6 pregnancies being affected [1]. GDM has long-term implications as women with a history of GDM have a 10-fold higher risk of developing type 2 diabetes (T2D) compared to those with a normoglycemic pregnancy [2]. In the Growing Up in Singapore Towards healthy Outcomes (GUSTO) study, women with GDM had a 12-fold higher risk of developing T2D 4-6 years after delivery compared with women who did not have GDM [3]. From a public health perspective, early intervention in women with GDM could contribute to tackling the rising global health burden of T2D. The T2D epidemic is of particular concern in Southeast Asia; 88 million adults are currently living with diabetes, but this is expected to increase to 153 million by 2045 [1]. Moreover, 57% of the population with diabetes in Southeast Asia are undiagnosed, increasing the risk of complications such as heart disease and stroke [1].

The American Diabetes Association guidelines recommend that women with GDM are tested 4-12 weeks postpartum using a 75 g oral glucose tolerance test (OGTT) [4]. Further testing is recommended in those with normal postpartum OGTT every 1-3 years using fasting plasma glucose, hemoglobin A_1c_ or HbA_1c_, or an OGTT [4]. However, as GDM resolves post pregnancy, postpartum surveillance of glycemia remains low across health care systems globally. The rate of postpartum diabetes screening can be as low as 13% in Asian countries [5]. Barriers to postpartum diabetes screening include lack of structured postpartum preventive care in health care systems, lack of patient awareness of future T2D risk, and time restrictions due to maternal commitments [5,6].

Machine learning models enable predictive population risk stratification. In a prospective metabolomics study by Allalou et al [7], 21 metabolites were identified at 6-9 weeks post partum to predict the transition from GDM to T2D in women. The metabolite model using decision trees performed well with an area under the receiver operating characteristic curve (AUC) of 0.77. In another GDM to T2D transition study by Joglekar et al [8], the inclusion of circulating microRNA (miR-369-3p) at 12 weeks post partum enhanced the prediction of a clinical model (age, BMI, pregnancy fasting glucose, postpartum fasting glucose, cholesterol, and triacylglycerols) from an AUC of 0.83 to an AUC of 0.92 (logistic regression algorithm). In addition to low compliance of postpartum testing in women with GDM, the other barriers to the real-world implementation of these 2 machine learning models include the cost and access to metabolomics assay and microRNA polymerase chain reaction during routine clinical visits.

The early prediction of postpartum T2D during prenatal care would enable the implementation of effective strategies for diabetes prevention interventions. To date, there have been no studies on using machine learning for postpartum T2D risk assessment in antenatal populations of Asian origin. In this study from Singapore, we developed a machine learning model for early prediction of postpartum T2D during routine antenatal GDM screening. Our machine learning model was implemented using the prospective GUSTO cohort study data (NCT01174875).

## Methods

### Ethics Approval

This study has been reviewed by the National Healthcare Group Domain Specific Review Board for ethics approval and SingHealth Centralized Institutional Review Board (CIRB/E/2019/2655).

### Study Design

GUSTO is a prospective multiethnic (Chinese, Malay, and Indian ethnicities) mother-offspring cohort study. Mothers were recruited during early pregnancy from Singapore’s 2 major public maternity hospitals, National University Hospital and KK Women’s and Children’s Hospital, between June 2009 and October 2010.

Participants of mixed ethnicity or with self-reported T2D at recruitment were excluded from model training. A total of 561 mothers had complete data on demographics, medical or obstetric history, physical measures, lifestyle information, antenatal OGTT, and postpartum OGTT 4-8 years after delivery. The World Health Organization (WHO) 1999 criteria [9] were used to diagnose GDM, and the WHO 2006 criteria [10] were used to diagnose postpartum impaired glucose tolerance (IGT), impaired fasting glucose (IFG), and T2D. The abnormal glucose metabolism (AGM) outcome comprises of IGT, IFG, and T2D diagnoses.

### Feature Variables

Information on demographics (maternal age, maternal ethnicity) and medical or obstetric history (self-reported prepregnancy weight, family history of diabetes mellitus, family history of high blood pressure, family history of cardiovascular disease, previous history of GDM, previous history of gestational hypertension, and parity) were derived from first trimester questionnaires. Systolic and diastolic blood pressure were recorded at midgestation (median 26.7, IQR 26.1-27.6 weeks) and obtained from hospital case notes. Mean arterial blood pressure was derived by doubling the diastolic blood pressure and adding to the systolic blood pressure, with the composite sum divided by 3. Maternal anthropometry was measured at midgestation (median 26.9, IQR 26.4-27.6 weeks). Maternal midupper arm circumference was measured to the nearest 0.1 cm, midway between acromion process and olecranon process (using Seca 212). Maternal height was measured to the nearest 0.1 cm (using Seca 213). Maternal weight at midpregnancy was measured to the nearest 0.1 kg (using Seca 803), and BMI was derived using weight divided by height squared (kg/m^2^). Total gestational weight gain was derived by subtracting first antenatal visit weight (median 9.0, IQR 7.3-11.0 weeks) from the last antenatal visit weight (median 38.1, IQR 37.3-39.1 weeks). Lifestyle information on self-reported smoking, environmental tobacco smoke exposures, and alcohol consumption were collected using questionnaires. GDM diagnosis was based on antenatal OGTT assessment (median 26.9, IQR 26.4-27.7 weeks).

### Machine Learning Methodology and Statistical Analyses

Our methodological novelty lies in combining coalitional game theory concepts with machine learning. SHapley Additive exPlanations (SHAP) framework was combined with CatBoost tree ensembles for feature selection and model explainability [11,12]. The SHAP framework connects optimal credit allocation with local explanations using the classic Shapley values from cooperative game theory. Lundberg and Lee [11] have proposed SHAP as the only additive feature attribution method that satisfies 2 important properties of game theory—additivity (local accuracy) and monotonicity (consistency). In game theory, Shapley value is the average expected marginal contribution of 1 player across all possible permutation of players (ie, the average effects of team member composition and team size). Shapley value helps determine a payoff for all the game players when each player might have contributed more or less than the others when working in coalition. In machine learning, the game players are the features, and the collective payout is the model prediction. SHAP framework provides local explanations based on exact Shapley values to understand the global model structure. For each possible feature ordering, features are introduced one at a time into a conditional expectation function of the model’s output, and changes in expectation are attributed to the introduced feature, averaged over all possible feature orderings in a fair manner. SHAP values represent a change in log odds ratio. Our game theoretical approach for predictive analytics enables population subtyping and pattern discovery for data-driven precision care.

The supervised machine learning models were built using Anaconda distribution of Python programming language (version 3.7.9) in JupyterLab computational environment. The predictive models were trained using the following 4 machine learning algorithms to address algorithm bias: logistic regression (generalized linear model), support vector machine (linear support vector classification), CatBoost gradient boosting (tree-based), and artificial neural network (multilayer perceptron). We used 5-fold stratified cross-validation to preserve the same proportion of AGM/T2D cases in each fold. Maximum absolute scaler was used as a preprocessor to scale each feature without destroying the sparsity. A grid search pipeline was built to evaluate the best performing hyperparameters for each machine learning model. Model performances were evaluated using the AUC with 95% CI. Implementation details of the machine learning algorithms are included in Multimedia Appendix 1.

The feature selection model using clinical features at midgestation was trained on the AGM outcome, and top predictors with SHAP value magnitudes more than zero were included in the AGM/T2D prediction models. Sensitivity analyses were performed to explore the prediction effects of diagnosing GDM using modified 2-point International Association of the Diabetes and Pregnancy Study Groups (IADPSG) 2018 criteria [9] rather than WHO 1999 criteria (GUSTO study did not include a 1-hour glucose measurement), and the prediction effects of continuous fasting or 2-hour glucose measures and prepregnancy BMI. We also assessed the associations between total gestational weight gain and postpartum AGM and T2D outcomes. All association analyses were performed using Stata/MP software (version 16.1; StataCorp LP).

## Results

### The Features Significantly Associated With T2D Aligned With the Top Features From the SHAP Feature Selection Model

**Figure 1:**
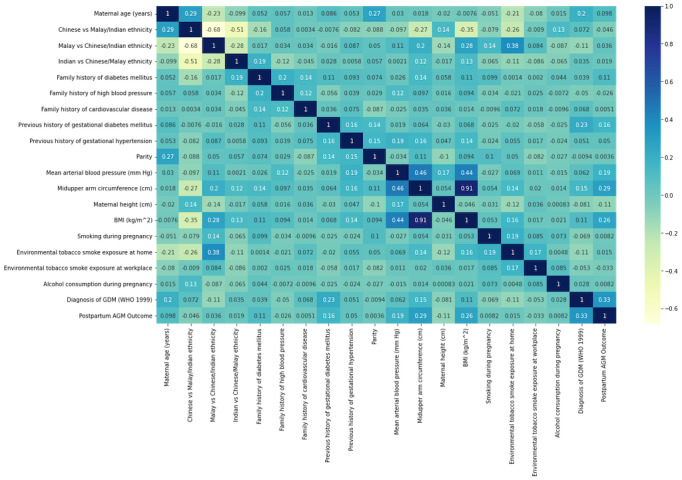
Pearson Correlation heatmap for abnormal glucose metabolism (AGM). GDM: gestational diabetes mellitus.

**Figure 2:**
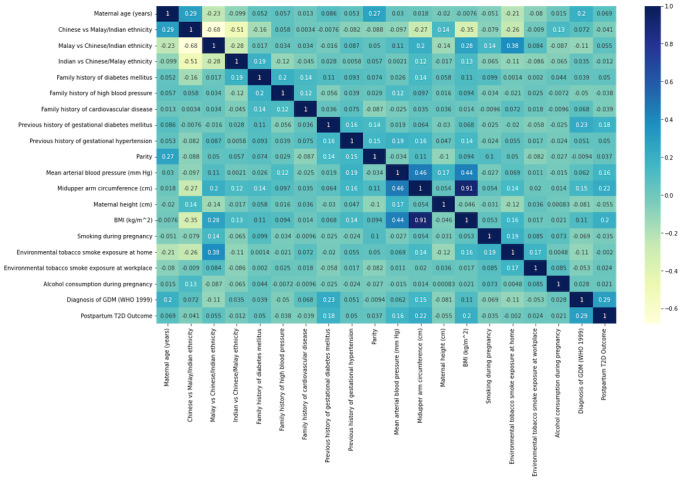
Pearson Correlation heatmap for type 2 diabetes (T2D). GDM: gestational diabetes mellitus.

The relationship between all feature variables and postpartum AGM and T2D outcomes is presented in a Pearson correlation heatmap (Figures 1 and 2). Diagnosis of GDM, midupper arm circumference, and BMI are the best features for postpartum AGM/T2D machine learning model building.

**Figure 3:**
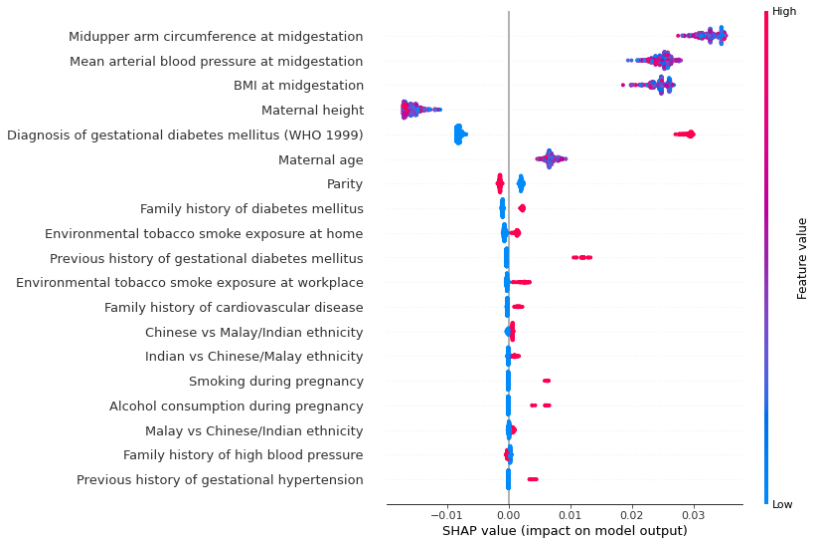
SHapley Additive exPlanations (SHAP) summery plot of feature selection model. WHO: World Health Organization.

Table 1 presents the univariate associations between midpregnancy features and postpartum AGM and T2D outcomes. Previous history of GDM, mean arterial blood pressure, midupper arm circumference, BMI, and diagnosis of GDM were associated with later risk of T2D. The top 4 features impacting the SHAP model outputs were midupper arm circumference, mean arterial blood pressure, BMI and diagnosis of GDM (Figure 3). The negative SHAP value for height implies that maternal height did not contribute to the prediction of AGM.

**Table 1:** Associations between midpregnancy characteristics and postpartum abnormal glucose metabolism (AGM) or type 2 diabetes (T2D) outcomes (4-8 years after delivery).

Characteristics	AGM (n=139)			T2D (n=32)	
	OR^a^ (95% CI)	P value	OR (95% CI)		P value
Maternal age (years)	1.05 (1.01-1.09)	.02^b^	1.06 (0.99-1.14)		.10
Chinese vs Malay and Indian ethnicity	0.81 (0.55-1.19)	.28	0.71 (0.34-1.44)		.34
Malay vs Chinese and Indian ethnicity	1.20 (0.79-1.83)	.40	1.64 (0.78-3.43)		.19
Indian vs Chinese and Malay ethnicity	1.12 (0.68-1.84)	.66	0.87 (0.33-2.31)		.78
Family history of diabetes mellitus	1.72 (1.15-2.56)	.008^b^	1.55 (0.75-3.21)		.24
Family history of high blood pressure	0.88 (0.60-1.32)	.55	0.70 (0.33-1.51)		.37
Family history of cardiovascular disease	1.04 (0.57-1.90)	.90	0.51 (0.12-2.19)		.37
Previous history of gestational diabetes mellitus	5.96 (2.16-16.43)	.001^b^	7.98 (2.62-24.27)		<.001^b^
Previous history of gestational hypertension	1.86 (0.66-5.21)	.24	2.45 (0.53-11.29)		.25
Parity	1.02 (0.69-1.50)	.93	1.38 (0.66-2.89)		.39
Mean arterial blood pressure (mm Hg)	1.05 (1.03-1.07)	<.001^b^	1.07 (1.03-1.11)		<.001^b^
Midupper arm circumference (cm)	1.18 (1.12-1.25)	<.001^b^	1.23 (1.13-1.33)		<.001^b^
Maternal height (cm)	0.96 (0.92-0.99)	.01^b^	0.96 (0.90-1.02)		.10
BMI (kg/m^2^)	1.14 (1.09-1.18)	<.001^b^	1.16 (1.09-1.24)		<.001^b^
Smoking during pregnancy	1.14 (0.30-4.36)	.85	N/A^c^		N/A
Environmental tobacco smoke exposure at home	1.07 (0.72-1.60)	.73	0.98 (0.46-2.08)		.96
Environmental tobacco smoke exposure at workplace	0.76 (0.38-1.51)	.43	1.37 (0.46-4.06)		.57
Alcohol consumption during pregnancy	1.14 (0.30-4.36)	.85	1.67 (0.21-13.50)		.63
Diagnosis of GDM^d^ (WHO^e^ 1999 criteria)	5.49 (3.51-8.58)	<.001^b^	9.57 (4.45-20.55)		<.001^b^

^a^OR: odds ratio.

^b^Indicates statistically significant values.

^c^N/A: not applicable; fixed-effect regression estimates were not obtained as the variable did not contribute to the likelihood estimation.

^d^GDM: gestational diabetes mellitus.

^e^WHO: World Health Organization.

### Maternal Adiposity During Pregnancy and Metabolic Derangements Underlying GDM Signaling Future T2D Risk

Although the detailed training parameters and results for all machine learning models are shown in Tables S1-S6 (Multimedia Appendix 2), we focus on describing the results of CatBoost machine learning models as this algorithm had the best overall performance. The results for each data set of the 5-fold stratified cross-validation and the average of the cross-validation are also provided in Tables S1-S6 in Multimedia Appendix 2. Midupper arm circumference at midgestation (AUC=0.78, 95% CI 0.71-0.86) and BMI at midgestation (AUC=0.74, 95% CI 0.53-0.96) had stronger predictive performances than GDM diagnosis (AUC=0.73, 95% CI 0.51-0.95; Table S2 in Multimedia Appendix 2). The addition of GDM diagnosis improved the performance of baseline models (MUAC_GDM model: AUC=0.88, 95% CI 0.79-0.96 and BMI_GDM model: AUC=0.86, 95% CI 0.72-0.99; Table S4 in Multimedia Appendix 2). Prepregnancy BMI alone was inadequate in predicting postpartum T2D risk (AUC=0.62, 95% CI 0.39-0.86; Table S6 in Multimedia Appendix 2).

**Figure 4:**
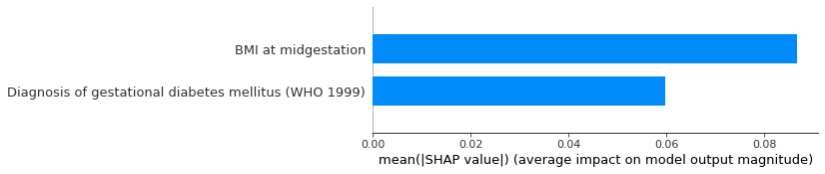
SHapley Additive exPlanations (SHAP) summary plot of BMI_GDM model. WHO: World Health Organization.

Although there is a high correlation between midupper arm circumference and BMI (*r*=0.91), BMI is more reliably and commonly assessed in clinical settings, and therefore, a BMI-based pregnancy model is our proposed solution (Figure 4). Table 2 summarizes the detailed training parameters of logistic regression, support vector machine, artificial neural network, and CatBoost gradient boosting algorithms, as well as the results of the proposed postpartum T2D predictive model (comprising of midpregnancy BMI after gestational weight gain and diagnosis of GDM features). Total gestational weight gain was inversely associated with postpartum AGM and T2D outcomes, independent of prepregnancy BMI and diagnosis of GDM (Table 3).

**Figure 5:**
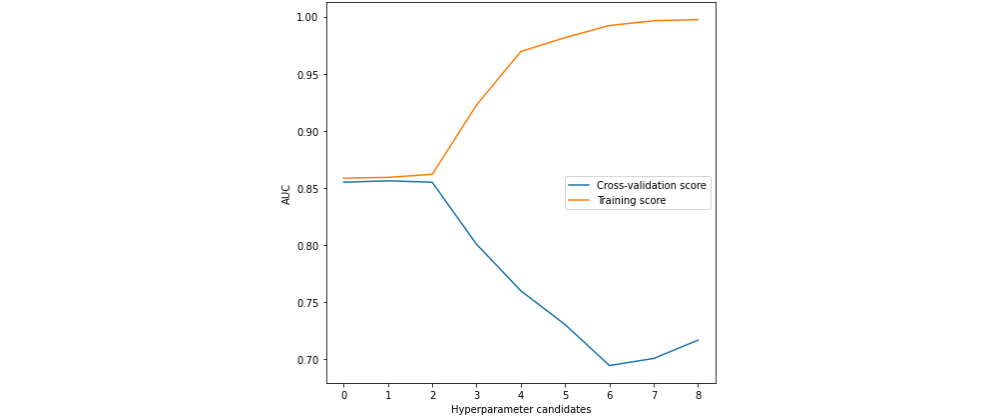
Validation curve with CatBoost algorithm–Varying learning rate. AUC: area under the receiver operating characteristic curve.

**Figure 6:**
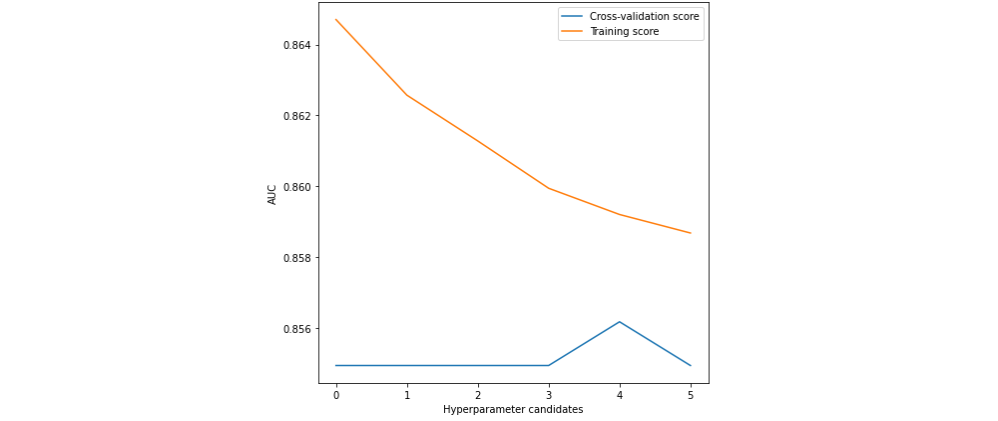
Validation curve with CatBoost algorithm–Varying L2 leaf regularization. AUC: area under the receiver operating characteristic curve.

**Figure 7:**
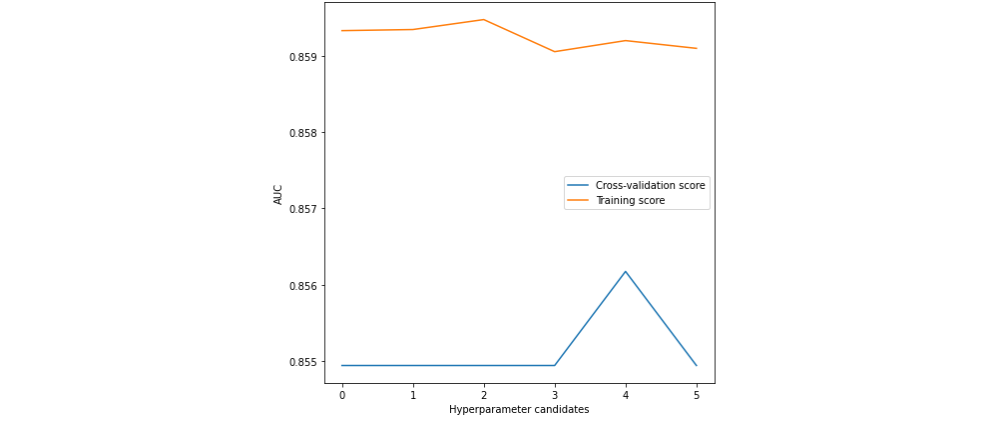
Validation curve with CatBoost algorithm–Varying random strength. AUC: area under the receiver operating characteristic curve.

Figures 5-7 present the validation curves obtained during the training of BMI_GDM CatBoost model. The hyperparameter candidates for CatBoost model were as follows:

- Learning rate: [‘0’ - 0.00001, **‘1’- 0.0001**, ‘2’ - 0.001, ‘3’ - 0.01, ‘4’ - 0.03, ‘5’ - 0.05, ‘6’ - 0.1, ‘7’ - 0.2, ‘8’ - 0.3]
- L2 leaf regularization: [‘0’ - 1.0, ‘1’ - 2.0, ‘2’ - 3.0, ‘3’ - 4.0, **‘4’ - 5.0**, ‘5’ - 6.0]
- Random strength: [‘0’ - 1.0, ‘1’ - 2.0, ‘2’ - 3.0, ‘3’ - 4.0, **‘4’ - 5.0**, ‘5’ - 6.0]

The CatBoost model was specified with 1000 iterations, maximum depth of 6 trees, and symmetric tree growing policy. The hyperparameters tuned using grid search were learning rate of 0.0001, L2 leaf regularization of 5.0, and random strength of 5.0. The BMI_GDM CatBoost classifier is performing well under this optimal configuration.

**Table 2:** Proposed postpartum type 2 diabetes predictive model comprising of midpregnancy BMI after gestational weight gain and diagnosis of gestational diabetes mellitus (GDM) features (based on the World Health Organization 1999 criteria).

Model specifications (BMI_GDM)	Hyperparameters tuned using grid search	Average AUC^a^ (95% CI)
Logistic regression (L2 regularization penalty, stochastic average gradient descent solver)	Inverse of regularization strength=1.0	0.85 (0.72-0.98)
Support vector machine (linear kernel, L2 regularization penalty)	L2 regularization penalty=1.0 Loss function=‘squared hinge’	0.85 (0.72-0.98)
Neural network (3 hidden layers with 10 neurons each, ReLU activation function, Adam solver, 200 iterations)	L2 regularization penalty=0.01 Initial learning rate=0.1	0.85 (0.73-0.97)
CatBoost^b^ (1000 iterations, maximum depth of 6 trees, symmetric tree growing policy)	L2 leaf regularization=5.0 Learning rate=0.0001 Random Strength=5.0	0.86 (0.72-0.99)^b^

^a^AUC: area under the receiver operating characteristic curve.

^b^Indicates the main predictive model developed in this study.

**Table 3:** Association between total gestational weight gain and postpartum abnormal glucose metabolism (AGM) or type 2 diabetes (T2D) outcomes (4-8 years after delivery).

Analysis			AGM (n=128)				T2D (n=31)		
			OR^a^ (95% CI)		P value		OR (95% CI)		P value
**Unadjusted analysis**									
	Total gestational weight gain (kg)	0.87 (0.82-0.91)		<.001^b^		0.79 (0.72-0.87)		<.001^b^	
**Adjusted analysis^c^**									
	Total gestational weight gain (kg)	0.93 (0.87-0.98)		.01^b^		0.88 (0.79-0.98)		.02^b^	

^a^OR: odds ratio.

^b^Indicates statistically significant values.

^c^Adjusted based on maternal ethnicity, age, parity, family history of diabetes mellitus, prepregnancy BMI, and diagnosis of gestational diabetes mellitus.

### Two-Hour Postprandial Glucose as a Stronger Predictor of Postpartum T2D Risk Compared With Fasting Glucose

When modeling antenatal glucose measures as continuous features, a 2-hour postprandial glucose (AUC=0.86, 95% CI 0.76-0.96) showed a stronger postpartum T2D risk prediction effect compared to fasting glucose (AUC=0.76, 95% CI 0.61-0.91; Table S6 in Multimedia Appendix 2). In the sensitivity analysis, predictive performance of BMI_GDM model was also robust when using the modified 2-point IADPSG 2018 criteria (AUC=0.84, 95% CI 0.72-0.97; Table S6 in Multimedia Appendix 2).

## Discussion

### Principal Results

We have built an effective postpartum T2D predictive model by combining game theory–based feature selection with machine learning. SHAP values recovered predictive modeling features for optimal performance, aligning model interpretability with human intuition. Our BMI_GDM model achieved an excellent AUC of 0.86 with 2 midgestation features (BMI at midgestation and diagnosis of GDM by the WHO 1999 criteria) for an early prediction of postpartum T2D risk in a Singapore population. The model was also robust when using a modified 2-point IADPSG 2018 criteria for GDM diagnosis (AUC=0.84). The BMI_GDM machine learning model can be leveraged as a risk stratification tool during routine GDM screening to identify Asian women at high risk of developing T2D, enabling early intervention. The BMI_2hour model (AUC=0.86) can be an alternative design during clinical implementation if GDM diagnosis feature is unavailable for the patient. The trained classifier can be deployed using a web application that can allow clinicians to identify women at T2D risk and develop a postpartum management plan.

The 2-feature midpregnancy BMI model (AUC=0.86) performed better in postpartum T2D prediction than a preconception BMI model (AUC=0.62), implying that midgestational weight gain effects combined with the metabolic derangements underlying GDM and fetoplacental unit signal future T2D risk. As pregnancy has a diabetogenic effect on metabolism [13], further studies will be required to examine the metabolic adaptations in pregnancy and postpartum maternal metabolic health outcomes.

In our BMI_GDM model sensitivity analysis, we observed that the 2-hour antenatal OGTT glucose peak was associated with a stronger prediction of postpartum T2D (AUC=0.86) compared with the fasting glucose (AUC=0.76) in Singaporean women. Future studies with greater statistical power will be needed to confirm whether the postpartum T2D risk is heterogenous across different thresholds of glucose tolerance for GDM diagnostic criteria.

### Limitations

This study has some limitations due to the scarcity of longitudinal data. Postpartum OGTT at 4-12 weeks, and further testing in those with normal postpartum OGTT every 1-3 years were not administered in the GUSTO study, possibly underestimating the development of postdelivery dysglycemia to a certain extent and inducing bias. However, the mothers participating in GUSTO self-reported T2D status 2 years after delivery, and there were no self-reported T2D cases. Our prediction models were trained on a limited cohort of 561 pregnancies and require further validation using larger cohorts such as Electronic Health Record databases. A subcohort analyses by individual ethnic groups can be trained with larger data sets.

### Comparison With Prior Work

Our early implementation of T2D risk prediction algorithm during prenatal care enables early engagement of patients and remote monitoring, compared to existing molecular biomarker-based T2D risk prediction algorithms [7,8] developed for postpartum care. The 2 midgestation clinical features (midpregnancy BMI after gestational weight gain and diagnosis of GDM) discovered from our machine learning workflow are of low cost and easily accessible during routine antenatal GDM screening. The digital biomarkers identified from our work will guide antenatal research in preventing the progression of GDM to T2D.

### Conclusions

The key strength of our study lies in applying machine learning–based predictive analytics during prenatal care in the early prediction of postpartum T2D. This machine learning model can be leveraged as a risk stratification tool for preventive intervention.

## Appendix

Multimedia Appendix 1 Implementation details of machine learning algorithms.

Multimedia Appendix 2 Tables S1-S6; detailed training parameters and results for all machine learning models.

## Abbreviations

AGM: abnormal glucose metabolism
AUC: area under the receiver operating characteristic curve
GDM: gestational diabetes mellitus
GUSTO: Growing Up in Singapore Towards healthy Outcomes
IADPSG: International Association of Diabetes and Pregnancy Study Groups
IFG: impaired fasting glucose
IGT: impaired glucose tolerance
OGTT: oral glucose tolerance test
SHAP: SHapley Additive exPlanations
T2D: type 2 diabetes
WHO: World Health Organization

## Data Availability

The data that support the findings of this research are available from the corresponding authors upon reasonable request. The code generated to reproduce this research is available at GitHub [14].

The postpartum T2D predictive model (CatBoost algorithm) has been deployed into a web application [15].
