# Supplementary material for: Machine Learning–Derived Prenatal Predictive Risk Model to Guide Intervention and Prevent the Progression of Gestational Diabetes Mellitus to Type 2 Diabetes: Prediction Model Development Study
Source: JMIR Diabetes. 2022 Jul 5;7(3):e32366. doi: 10.2196/32366 (PMC9297138; doi:10.2196/32366)
Supplement: Multimedia Appendix 1 [file diabetes_v7i3e32366_app1.docx]

**Multimedia Appendix 1. Implementation of Machine Learning Algorithms**

Community-developed Python packages were used for modular programming; Pandas v0.25.3, Numpy v1.19.2, Matplotlib v3.3.2, Seaborn v0.11.2, Scikit-learn v0.23.2, CatBoost v0.16.2 and Shap v0.37.0. We trained the machine learning models in a Linux server with Intel Xeon Gold 6138 CPU processor.

**CatBoost Gradient Boosting**

CatBoost is a gradient boosting machine learning technique for learning problems with heterogenous features and complex dependencies. In gradient boosting, an ensemble predictor is built with binary decision trees by performing gradient descent in a functional space.

By using ordered boosting, CatBoost algorithm with default parameters outperforms XGBoost and LightGBM algorithms (existing state-of-the-art implementations of gradient boosted decision trees) on popular machine learning tasks [12]. In ordered boosting, a new dataset D_t_ is independently sampled at each step of boosting to avoid prediction shift. Unshifted residuals are obtained by applying the current model to new training examples [12]. CatBoost algorithm is less prone to overfitting and generally robust to model changes as the same splitting criterion is used across the full binary tree structure [12].

The CatBoost model was specified with 1000 iterations, maximum depth of 6 trees and symmetric tree growing policy. In model parameters, we tuned the L2 leaf regularization (penalizes the sum of square weights for a simpler model), learning rate (reduces the gradient step) and random strength (randomness for scoring splits in tree structure).

L2 leaf regularization: {1.0, 2.0, 3.0, 4.0, 5.0, 6.0}

Learning rate: {0.00001, 0.0001, 0.001, 0.01, 0.03, 0.05, 0.1, 0.2, 0.3}

Random strength: {1.0, 2.0, 3.0, 4.0, 5.0, 6.0}

**Logistic Regression**

Logistic regression is a generalized linear model which estimates the probability of T2D event occurrence by fitting data onto a logistic curve.

The logistic regression model was specified with L2 regularization penalty and stochastic average gradient descent solver. In model parameters, we tuned the inverse of regularization strength (for a simpler model).

Inverse of regularization strength: {1.0, 2.0, 3.0, 4.0, 5.0, 6.0, 7.0, 8.0, 9.0, 10.0}

**Support Vector Machine**

For a binary classification problem, support vector machine fits a decision boundary between training points from two different classes (T2D, Non-T2D). Support vector machine maximizes the margin (distance between a training example and decision boundary) to predict for each new test point, which of the two different classes it belongs to.

The support vector machine model was specified with linear kernel and L2 regularization penalty. In model parameters; we tuned the L2 regularization penalty (penalizes the sum of square weights for a simpler model) and hinge loss convex function.

L2 regularization penalty: {1.0, 2.0, 3.0, 4.0, 5.0, 6.0}

Loss function: {‘hinge’, ‘squared_hinge’}

**Artificial Neural Network**

We trained an artificial neural network (multilayer perceptron) that can learn a non-linear function approximator. In between the input and output layer, there are non-linear layers (hidden layers). The input layer consists of the input features. The neurons in the hidden layer transforms the values from previous layer with a weighted linear summation, followed by a non-linear activation function (e.g. ReLU function). The last hidden layer passes the values into output layer, transforming them into output values. Multi-layer perceptron trains using gradient descent and the gradients are calculated using backpropagation for 200 iterations. We configured 3 hidden layers with 10 neurons each. We also specified Rectified Linear Unit (ReLU) activation function with Adam solver for weight optimization.

In model parameters, we tuned the L2 regularization penalty (penalizes the sum of square weights for a simpler model) and initial learning rate (controls the step size in updating weights).

L2 regularization penalty: {0.00001, 0.0001, 0.001, 0.01, 0.1}

Initial learning rate: {0.00001, 0.0001, 0.001, 0.01, 0.1}

**Shapley values and SHapley Additive exPlanations (SHAP)**

Explainable AI technique, Shapley values and SHapley Additive exPlanations (SHAP) has its foundations in cooperative game theory. Lloyd Shapley was awarded the Nobel Memorial Prize in Economic Science for his work on Shapley value. The Nobel Prize winning method is being used by Google AI platform as a feature attribution technique.

**SHAP Summary Plot**

SHAP summary plots display the magnitude, prevalence and direction of feature effects. In the SHAP plot, local explanations are plotted in a beeswarm style to observe the magnitude and prevalence of a feature’s effect. Each dot corresponds to an individual subject. The colors display the direction of feature effects, red dots represent a high value of that feature for that subject and blue dots represent a low value of that feature. If the feature’s impact (value changes) varies smoothly on the model’s output, the coloring will have a smooth gradation.

Each subject has one dot for each feature - for that subject, the position of the dot on horizontal axis corresponds to the feature’s impact on model prediction. Multiple dots at the same position in the horizontal axis are piled up and shown as density (stacked vertically when they run out of space). The vertical stacking creates a violin-plot effect.

The average magnitude of the SHAP values is displayed in the horizonal axis and features are sorted by global impact.
