# Supplementary material for: Machine Learning–Derived Prenatal Predictive Risk Model to Guide Intervention and Prevent the Progression of Gestational Diabetes Mellitus to Type 2 Diabetes: Prediction Model Development Study
Source: JMIR Diabetes. 2022 Jul 5;7(3):e32366. doi: 10.2196/32366 (PMC9297138; doi:10.2196/32366)
Supplement: Multimedia Appendix 2 [file diabetes_v7i3e32366_app2.docx]

**Multimedia Appendix 2.** Tables S1-S6; detailed training parameters and results for all machine learning models.

**Supplementary Table S1: Single Feature Panel (AGM)**

| **Features: Mid-upper arm circumference at mid-gestation (MUAC)** | **Hyperparameters tuned using grid search** | **5-fold stratified cross validation scores of best estimator (AUC)** | **Average AUC (95% CI)** |
| --- | --- | --- | --- |
| **Model Specifications** |  |  |  |
| Logistic Regression (L2 regularization penalty, stochastic average gradient descent solver) | Inverse of regularization strength = 1.0 | Fold 1: 0.66943522  Fold 2: 0.56561462  Fold 3: 0.76328904  Fold 4: 0.80398671  Fold 5: 0.76162791 | 0.71 (0.55, 0.88) |
| Support Vector Machine (linear kernel, L2 regularization penalty) | L2 regularization penalty = 1.0  Loss function = ‘hinge’ | Fold 1: 0.66943522  Fold 2: 0.56561462  Fold 3: 0.76328904  Fold 4: 0.80398671  Fold 5: 0.76162791 | 0.71 (0.55, 0.88) |
| Neural Network (3 hidden layers with 10 neurons each, ReLU activation function, Adam solver, 200 iterations) | L2 regularization penalty = 0.001  Initial learning rate = 0.1 | Fold 1: 0.67441860  Fold 2: 0.56561462  Fold 3: 0.75830565  Fold 4: 0.80398671  Fold 5: 0.76162791 | 0.71 (0.55, 0.88) |
| **CatBoost** (1000 iterations, maximum depth of 6 trees, symmetric tree growing policy) | L2 leaf regularization = 5.0  Learning rate = 0.0001  Random Strength = 5.0 | Fold 1: 0.67607973  Fold 2: 0.55730897  Fold 3: 0.76910299  Fold 4: 0.78737542  Fold 5: 0.75913621 | **0.71 (0.54, 0.88)** |
| **Features: Diagnosis of gestational diabetes mellitus (WHO 1999) (GDM)** | **Hyperparameters tuned using grid search** | **5-fold stratified cross validation scores of best estimator (AUC)** | **Average AUC (95% CI)** |
| **Model Specifications** |  |  |  |
| Logistic Regression (L2 regularization penalty, stochastic average gradient descent solver) | Inverse of regularization strength = 1.0 | Fold 1: 0.63205980  Fold 2: 0.72757475  Fold 3: 0.56229236  Fold 4: 0.53820598  Fold 5: 0.71594684 | 0.64 (0.48, 0.79) |
| Support Vector Machine (linear kernel, L2 regularization penalty) | L2 regularization penalty = 1.0  Loss function = ‘hinge’ | Fold 1: 0.63205980  Fold 2: 0.72757475  Fold 3: 0.56229236  Fold 4: 0.53820598  Fold 5: 0.71594684 | 0.64 (0.48, 0.79) |
| Neural Network (3 hidden layers with 10 neurons each, ReLU activation function, Adam solver, 200 iterations) | L2 regularization penalty = 0.1  Initial learning rate = 0.001 | Fold 1: 0.63205980  Fold 2: 0.72757475  Fold 3: 0.56229236  Fold 4: 0.53820598  Fold 5: 0.71594684 | 0.64 (0.48, 0.79) |
| **CatBoost** (1000 iterations, maximum depth of 6 trees, symmetric tree growing policy) | L2 leaf regularization = 1.0  Learning rate = 0.00001  Random Strength = 1.0 | Fold 1: 0.63205980  Fold 2: 0.72757475  Fold 3: 0.56229236  Fold 4: 0.53820598  Fold 5: 0.71594684 | **0.64 (0.48, 0.79)** |
| **Features: BMI at mid-gestation (BMI)** | **Hyperparameters tuned using grid search** | **5-fold stratified cross validation scores of best estimator (AUC)** | **Average AUC (95% CI)** |
| **Model Specifications** |  |  |  |
| Logistic Regression (L2 regularization penalty, stochastic average gradient descent solver) | Inverse of regularization strength = 1.0 | Fold 1: 0.70681063  Fold 2: 0.56146179  Fold 3: 0.74584718  Fold 4: 0.82059801  Fold 5: 0.75581395 | 0.72 (0.55, 0.89) |
| Support Vector Machine (linear kernel, L2 regularization penalty) | L2 regularization penalty = 1.0  Loss function = ‘hinge’ | Fold 1: 0.70681063  Fold 2: 0.56146179  Fold 3: 0.74584718  Fold 4: 0.82059801  Fold 5: 0.75581395 | 0.72 (0.55, 0.89) |
| Neural Network (3 hidden layers with 10 neurons each, ReLU activation function, Adam solver, 200 iterations) | L2 regularization penalty = 0.01  Initial learning rate = 0.1 | Fold 1: 0.70681063  Fold 2: 0.56644518  Fold 3: 0.74584718  Fold 4: 0.82059801  Fold 5: 0.75581395 | 0.72 (0.55, 0.89) |
| **CatBoost** (1000 iterations, maximum depth of 6 trees, symmetric tree growing policy) | L2 leaf regularization = 5.0  Learning rate = 0.05  Random Strength = 4.0 | Fold 1: 0.73089701  Fold 2: 0.66196013  Fold 3: 0.82308970  Fold 4: 0.83720930  Fold 5: 0.74418605 | **0.76 (0.63, 0.89)** |
| **Features: Mean arterial blood pressure at mid-gestation (MAP)** | **Hyperparameters tuned using grid search** | **5-fold stratified cross validation scores of best estimator (AUC)** | **Average AUC (95% CI)** |
| **Model Specifications** |  |  |  |
| Logistic Regression (L2 regularization penalty, stochastic average gradient descent solver) | Inverse of regularization strength = 1.0 | Fold 1: 0.61794020  Fold 2: 0.52408638  Fold 3: 0.68023256  Fold 4: 0.77906977  Fold 5: 0.57973422 | 0.64 (0.46, 0.81) |
| Support Vector Machine (linear kernel, L2 regularization penalty) | L2 regularization penalty = 1.0  Loss function = ‘hinge’ | Fold 1: 0.61794020  Fold 2: 0.52408638  Fold 3: 0.68023256  Fold 4: 0.77906977  Fold 5: 0.57973422 | 0.64 (0.46, 0.81) |
| Neural Network (3 hidden layers with 10 neurons each, ReLU activation function, Adam solver, 200 iterations) | L2 regularization penalty = 0.00001  Initial learning rate = 0.01 | Fold 1: 0.61794020  Fold 2: 0.53571429  Fold 3: 0.68023256  Fold 4: 0.77906977  Fold 5: 0.57475083 | 0.64 (0.47, 0.81) |
| **CatBoost** (1000 iterations, maximum depth of 6 trees, symmetric tree growing policy) | L2 leaf regularization = 6.0  Learning rate = 0.00001  Random Strength = 5.0 | Fold 1: 0.62790698  Fold 2: 0.53073090  Fold 3: 0.66528239  Fold 4: 0.75083056  Fold 5: 0.58056478 | **0.63 (0.48, 0.78)** |

**Supplementary Table S2: Single Feature Panel (T2D)**

| **Features: Mid-upper arm circumference at mid-gestation (MUAC)** | **Hyperparameters tuned using grid search** | **5-fold stratified cross validation scores of best estimator (AUC)** | **Average AUC (95% CI)** |
| --- | --- | --- | --- |
| **Model Specifications** |  |  |  |
| Logistic Regression (L2 regularization penalty, stochastic average gradient descent solver) | Inverse of regularization strength = 1.0 | Fold 1: 0.73456790  Fold 2: 0.93209877  Fold 3: 0.80246914  Fold 4: 0.72839506  Fold 5: 0.59876543 | 0.76 (0.55, 0.97) |
| Support Vector Machine (linear kernel, L2 regularization penalty) | L2 regularization penalty = 1.0  Loss function = ‘hinge’ | Fold 1: 0.73456790  Fold 2: 0.93209877  Fold 3: 0.80246914  Fold 4: 0.72839506  Fold 5: 0.59876543 | 0.76 (0.55, 0.97) |
| Neural Network (3 hidden layers with 10 neurons each, ReLU activation function, Adam solver, 200 iterations) | L2 regularization penalty = 0.01  Initial learning rate = 0.1 | Fold 1: 0.72222222  Fold 2: 0.93209877  Fold 3: 0.80246914  Fold 4: 0.72839506  Fold 5: 0.59876543 | 0.76 (0.55, 0.97) |
| **CatBoost** (1000 iterations, maximum depth of 6 trees, symmetric tree growing policy) | L2 leaf regularization = 3.0  Learning rate = 0.01  Random Strength = 1.0 | Fold 1: 0.73456790  Fold 2: 0.84876543  Fold 3: 0.76543210  Fold 4: 0.79629630  Fold 5: 0.76543210 | **0.78 (0.71, 0.86)** |
| **Features: Diagnosis of gestational diabetes mellitus (WHO 1999) (GDM)** | **Hyperparameters tuned using grid search** | **5-fold stratified cross validation scores of best estimator (AUC)** | **Average AUC (95% CI)** |
| **Model Specifications** |  |  |  |
| Logistic Regression (L2 regularization penalty, stochastic average gradient descent solver) | Inverse of regularization strength = 1.0 | Fold 1: 0.92592593  Fold 2: 0.68518519  Fold 3: 0.69444444  Fold 4: 0.59259259  Fold 5: 0.75000000 | 0.73 (0.51, 0.95) |
| Support Vector Machine (linear kernel, L2 regularization penalty) | L2 regularization penalty = 1.0  Loss function = ‘hinge’ | Fold 1: 0.92592593  Fold 2: 0.68518519  Fold 3: 0.69444444  Fold 4: 0.59259259  Fold 5: 0.75000000 | 0.73 (0.51, 0.95) |
| Neural Network (3 hidden layers with 10 neurons each, ReLU activation function, Adam solver, 200 iterations) | L2 regularization penalty = 0.001  Initial learning rate = 0.1 | Fold 1: 0.92592593  Fold 2: 0.68518519  Fold 3: 0.69444444  Fold 4: 0.59259259  Fold 5: 0.75000000 | 0.73 (0.51, 0.95) |
| **CatBoost** (1000 iterations, maximum depth of 6 trees, symmetric tree growing policy) | L2 leaf regularization = 1.0  Learning rate = 0.00001  Random Strength = 1.0 | Fold 1: 0.92592593  Fold 2: 0.68518519  Fold 3: 0.69444444  Fold 4: 0.59259259  Fold 5: 0.75000000 | **0.73 (0.51, 0.95)** |
| **Features: BMI at mid-gestation (BMI)** | **Hyperparameters tuned using grid search** | **5-fold stratified cross validation scores of best estimator (AUC)** | **Average AUC (95% CI)** |
| **Model Specifications** |  |  |  |
| Logistic Regression (L2 regularization penalty, stochastic average gradient descent solver) | Inverse of regularization strength = 1.0 | Fold 1: 0.75308642  Fold 2: 0.89506173  Fold 3: 0.87037037  Fold 4: 0.68209877  Fold 5: 0.54320988 | 0.75 (0.50, 0.99) |
| Support Vector Machine (linear kernel, L2 regularization penalty) | L2 regularization penalty = 1.0  Loss function = ‘hinge’ | Fold 1: 0.75308642  Fold 2: 0.89506173  Fold 3: 0.87037037  Fold 4: 0.68209877  Fold 5: 0.54320988 | 0.75 (0.50, 0.99) |
| Neural Network (3 hidden layers with 10 neurons each, ReLU activation function, Adam solver, 200 iterations) | L2 regularization penalty = 0.001  Initial learning rate = 0.1 | Fold 1: 0.75308642  Fold 2: 0.89506173  Fold 3: 0.87037037  Fold 4: 0.68209877  Fold 5: 0.54320988 | 0.75 (0.50, 0.99) |
| **CatBoost** (1000 iterations, maximum depth of 6 trees, symmetric tree growing policy) | L2 leaf regularization = 5.0  Learning rate = 0.00001  Random Strength = 6.0 | Fold 1: 0.74074074  Fold 2: 0.87037037  Fold 3: 0.84876543  Fold 4: 0.68518519  Fold 5: 0.57716049 | **0.74 (0.53, 0.96)** |
| **Features: Mean arterial blood pressure at mid-gestation (MAP)** | **Hyperparameters tuned using grid search** | **5-fold stratified cross validation scores of best estimator (AUC)** | **Average AUC (95% CI)** |
| **Model Specifications** |  |  |  |
| Logistic Regression (L2 regularization penalty, stochastic average gradient descent solver) | Inverse of regularization strength = 1.0 | Fold 1: 0.54629630  Fold 2: 0.61111111  Fold 3: 0.69753086  Fold 4: 0.66049383  Fold 5: 0.77160494 | 0.66 (0.51, 0.81) |
| Support Vector Machine (linear kernel, L2 regularization penalty) | L2 regularization penalty = 1.0  Loss function = ‘hinge’ | Fold 1: 0.54629630  Fold 2: 0.61111111  Fold 3: 0.69753086  Fold 4: 0.66049383  Fold 5: 0.77160494 | 0.66 (0.51, 0.81) |
| Neural Network (3 hidden layers with 10 neurons each, ReLU activation function, Adam solver, 200 iterations) | L2 regularization penalty = 0.01  Initial learning rate = 0.1 | Fold 1: 0.54629630  Fold 2: 0.61111111  Fold 3: 0.30246914  Fold 4: 0.66049383  Fold 5: 0.77469136 | 0.66 (0.51, 0.81) |
| **CatBoost** (1000 iterations, maximum depth of 6 trees, symmetric tree growing policy) | L2 leaf regularization = 6.0  Learning rate = 0.00001  Random Strength = 4.0 | Fold 1: 0.47222222  Fold 2: 0.77777778  Fold 3: 0.56172840  Fold 4: 0.62037037  Fold 5: 0.79629630 | **0.65 (0.40, 0.89)** |

**Supplementary Table S3: Multiple Feature Panel (AGM)**

| **Features: Mid-upper arm circumference at mid-gestation + Diagnosis of gestational diabetes mellitus (WHO 1999) (MUAC_GDM)** | **Hyperparameters tuned using grid search** | **5-fold stratified cross validation scores of best estimator (AUC)** | **Average AUC (95% CI)** |
| --- | --- | --- | --- |
| **Model Specifications** |  |  |  |
| Logistic Regression (L2 regularization penalty, stochastic average gradient descent solver) | Inverse of regularization strength = 5.0 | Fold 1: 0.74501661  Fold 2: 0.72009967  Fold 3: 0.68687708  Fold 4: 0.73920266  Fold 5: 0.82973422 | 0.74 (0.65, 0.84) |
| Support Vector Machine (linear kernel, L2 regularization penalty) | L2 regularization penalty = 3.0  Loss function = ‘squared hinge’ | Fold 1: 0.74501661  Fold 2: 0.72009967  Fold 3: 0.68687708  Fold 4: 0.73920266  Fold 5: 0.82973422 | 0.74 (0.65, 0.84) |
| Neural Network (3 hidden layers with 10 neurons each, ReLU activation function, Adam solver, 200 iterations) | L2 regularization penalty = 0.01  Initial learning rate = 0.1 | Fold 1: 0.74501661  Fold 2: 0.72176080  Fold 3: 0.69019934  Fold 4: 0.73006645  Fold 5: 0.82308970 | 0.74 (0.66, 0.83) |
| **CatBoost** (1000 iterations, maximum depth of 6 trees, symmetric tree growing policy) | L2 leaf regularization = 3.0  Learning rate = 0.001  Random Strength = 4.0 | Fold 1: 0.74335548  Fold 2: 0.73421927  Fold 3: 0.68936877  Fold 4: 0.73920266  Fold 5: 0.82890365 | **0.75 (0.66, 0.84)** |
| **Features: BMI at mid-gestation + Diagnosis of gestational diabetes mellitus (WHO 1999) (BMI_GDM)** | **Hyperparameters tuned using grid search** | **5-fold stratified cross validation scores of best estimator (AUC)** | **Average AUC (95% CI)** |
| **Model Specifications** |  |  |  |
| Logistic Regression (L2 regularization penalty, stochastic average gradient descent solver) | Inverse of regularization strength = 2.0 | Fold 1: 0.77990033  Fold 2: 0.74252492  Fold 3: 0.68770764  Fold 4: 0.75083056  Fold 5: 0.81893688 | 0.76 (0.67, 0.84) |
| Support Vector Machine (linear kernel, L2 regularization penalty) | L2 regularization penalty = 1.0  Loss function = ‘squared hinge’ | Fold 1: 0.77990033  Fold 2: 0.73920266  Fold 3: 0.68770764  Fold 4: 0.75083056  Fold 5: 0.82059801 | 0.76 (0.67, 0.84) |
| Neural Network (3 hidden layers with 10 neurons each, ReLU activation function, Adam solver, 200 iterations) | L2 regularization penalty = 0.01  Initial learning rate = 0.01 | Fold 1: 0.78322259  Fold 2: 0.72591362  Fold 3: 0.69102990  Fold 4: 0.75083056  Fold 5: 0.82059801 | 0.76 (0.67, 0.84) |
| **CatBoost** (1000 iterations, maximum depth of 6 trees, symmetric tree growing policy) | L2 leaf regularization = 5.0  Learning rate = 0.3  Random Strength = 6.0 | Fold 1: 0.78571429  Fold 2: 0.80232558  Fold 3: 0.77159468  Fold 4: 0.80647841  Fold 5: 0.74335548 | **0.78 (0.74, 0.83)** |
| **Features: Mean arterial blood pressure at mid-gestation + Diagnosis of gestational diabetes mellitus (WHO 1999) (MAP_GDM)** | **Hyperparameters tuned using grid search** | **5-fold stratified cross validation scores of best estimator (AUC)** | **Average AUC (95% CI)** |
| **Model Specifications** |  |  |  |
| Logistic Regression (L2 regularization penalty, stochastic average gradient descent solver) | Inverse of regularization strength = 9.0 | Fold 1: 0.72840532  Fold 2: 0.73338870  Fold 3: 0.63039867  Fold 4: 0.71345515  Fold 5: 0.75249169 | 0.71 (0.63, 0.80) |
| Support Vector Machine (linear kernel, L2 regularization penalty) | L2 regularization penalty = 6.0  Loss function = ‘squared hinge’ | Fold 1: 0.72840532  Fold 2: 0.73338870  Fold 3: 0.63039867  Fold 4: 0.71345515  Fold 5: 0.75083056 | 0.71 (0.63, 0.79) |
| Neural Network (3 hidden layers with 10 neurons each, ReLU activation function, Adam solver, 200 iterations) | L2 regularization penalty = 0.0001  Initial learning rate = 0.1 | Fold 1: 0.72840532  Fold 2: 0.73338870  Fold 3: 0.58139535  Fold 4: 0.71345515  Fold 5: 0.74252492 | 0.71 (0.63, 0.79) |
| **CatBoost** (1000 iterations, maximum depth of 6 trees, symmetric tree growing policy) | L2 leaf regularization = 2.0  Learning rate = 0.001  Random Strength = 5.0 | Fold 1: 0.73172757  Fold 2: 0.73671096  Fold 3: 0.63704319  Fold 4: 0.71677741  Fold 5: 0.73671096 | **0.71 (0.64, 0.79)** |
| **Features: Mid-upper arm circumference at mid-gestation + BMI at mid-gestation + Mean arterial blood pressure at mid-gestation Diagnosis of gestational diabetes mellitus (WHO 1999) (MUAC_BMI_MAP_GDM)** | **Hyperparameters tuned using grid search** | **5-fold stratified cross validation scores of best estimator (AUC)** | **Average AUC (95% CI)** |
| **Model Specifications** |  |  |  |
| Logistic Regression (L2 regularization penalty, stochastic average gradient descent solver) | Inverse of regularization strength = 1.0 | Fold 1: 0.76411960  Fold 2: 0.72923588  Fold 3: 0.69601329  Fold 4: 0.76411960  Fold 5: 0.82558140 | 0.76 (0.67, 0.84) |
| Support Vector Machine (linear kernel, L2 regularization penalty) | L2 regularization penalty = 1.0  Loss function = ‘squared hinge’ | Fold 1: 0.76411960  Fold 2: 0.72093023  Fold 3: 0.70265781  Fold 4: 0.75581395  Fold 5: 0.82392027 | 0.75 (0.67, 0.84) |
| Neural Network (3 hidden layers with 10 neurons each, ReLU activation function, Adam solver, 200 iterations) | L2 regularization penalty = 0.01  Initial learning rate = 0.1 | Fold 1: 0.77408638  Fold 2: 0.72591362  Fold 3: 0.70265781  Fold 4: 0.74584718  Fold 5: 0.82225914 | 0.76 (0.67, 0.84) |
| **CatBoost** (1000 iterations, maximum depth of 6 trees, symmetric tree growing policy) | L2 leaf regularization = 3.0  Learning rate = 0.001  Random Strength = 6.0 | Fold 1: 0.75415282  Fold 2: 0.75249169  Fold 3: 0.72093023  Fold 4: 0.76744186  Fold 5: 0.79900332 | **0.76 (0.71, 0.81)** |

**Supplementary Table S4: Multiple Feature Panel (T2D)**

| **Features: Mid-upper arm circumference at mid-gestation + Diagnosis of gestational diabetes mellitus (WHO 1999) (MUAC_GDM)** | **Hyperparameters tuned using grid search** | **5-fold stratified cross validation scores of best estimator (AUC)** | **Average AUC (95% CI)** |
| --- | --- | --- | --- |
| **Model Specifications** |  |  |  |
| Logistic Regression (L2 regularization penalty, stochastic average gradient descent solver) | Inverse of regularization strength = 1.0 | Fold 1: 0.93209877  Fold 2: 0.87037037  Fold 3: 0.82407407  Fold 4: 0.78395062  Fold 5: 0.84567901 | 0.85 (0.75, 0.95) |
| Support Vector Machine (linear kernel, L2 regularization penalty) | L2 regularization penalty = 5.0  Loss function = ‘squared hinge’ | Fold 1: 0.93209877  Fold 2: 0.87037037  Fold 3: 0.84876543  Fold 4: 0.78395062  Fold 5: 0.83950617 | 0.86 (0.76, 0.95) |
| Neural Network (3 hidden layers with 10 neurons each, ReLU activation function, Adam solver, 200 iterations) | L2 regularization penalty = 0.01  Initial learning rate = 0.1 | Fold 1: 0.93209877  Fold 2: 0.86419753  Fold 3: 0.81481481  Fold 4: 0.78395062  Fold 5: 0.83950617 | 0.86 (0.76, 0.96) |
| **CatBoost** (1000 iterations, maximum depth of 6 trees, symmetric tree growing policy) | L2 leaf regularization = 3.0  Learning rate = 0.01  Random Strength = 3.0 | Fold 1: 0.91975309  Fold 2: 0.85802469  Fold 3: 0.85493827  Fold 4: 0.80864198  Fold 5: 0.93209877 | **0.88 (0.79, 0.96)** |
| **Features: BMI at mid-gestation + Diagnosis of gestational diabetes mellitus (WHO 1999) (BMI_GDM)** | **Hyperparameters tuned using grid search** | **5-fold stratified cross validation scores of best estimator (AUC)** | **Average AUC (95% CI)** |
| **Model Specifications** |  |  |  |
| Logistic Regression (L2 regularization penalty, stochastic average gradient descent solver) | Inverse of regularization strength = 1.0 | Fold 1: 0.95061728  Fold 2: 0.85802469  Fold 3: 0.86419753  Fold 4: 0.74382716  Fold 5: 0.84567901 | 0.85 (0.72, 0.98) |
| Support Vector Machine (linear kernel, L2 regularization penalty) | L2 regularization penalty = 1.0  Loss function = ‘squared hinge’ | Fold 1: 0.95061728  Fold 2: 0.85802469  Fold 3: 0.86419753  Fold 4: 0.74382716  Fold 5: 0.83950617 | 0.85 (0.72, 0.98) |
| Neural Network (3 hidden layers with 10 neurons each, ReLU activation function, Adam solver, 200 iterations) | L2 regularization penalty = 0.01  Initial learning rate = 0.1 | Fold 1: 0.94135802  Fold 2: 0.85185185  Fold 3: 0.86419753  Fold 4: 0.74382716  Fold 5: 0.82716049 | 0.85 (0.73, 0.97) |
| **CatBoost** (1000 iterations, maximum depth of 6 trees, symmetric tree growing policy) | L2 leaf regularization = 5.0  Learning rate = 0.0001  Random Strength = 5.0 | Fold 1: 0.95679012  Fold 2: 0.86419753  Fold 3: 0.86419753  Fold 4: 0.74382716  Fold 5: 0.85185185 | **0.86 (0.72, 0.99)** |
| **Features: Mean arterial blood pressure at mid-gestation + Diagnosis of gestational diabetes mellitus (WHO 1999) (MAP_GDM)** | **Hyperparameters tuned using grid search** | **5-fold stratified cross validation scores of best estimator (AUC)** | **Average AUC (95% CI)** |
| **Model Specifications** |  |  |  |
| Logistic Regression (L2 regularization penalty, stochastic average gradient descent solver) | Inverse of regularization strength = 1.0 | Fold 1: 0.91975309  Fold 2: 0.77777778  Fold 3: 0.76543210  Fold 4: 0.80246914  Fold 5: 0.88271605 | 0.83 (0.71, 0.95) |
| Support Vector Machine (linear kernel, L2 regularization penalty) | L2 regularization penalty = 1.0  Loss function = ‘squared hinge’ | Fold 1: 0.91975309  Fold 2: 0.77777778  Fold 3: 0.76543210  Fold 4: 0.80246914  Fold 5: 0.88271605 | 0.83 (0.71, 0.95) |
| Neural Network (3 hidden layers with 10 neurons each, ReLU activation function, Adam solver, 200 iterations) | L2 regularization penalty = 0.001  Initial learning rate = 0.1 | Fold 1: 0.92592593  Fold 2: 0.76543210  Fold 3: 0.76543210  Fold 4: 0.37037037  Fold 5: 0.87037037 | 0.82 (0.68, 0.96) |
| **CatBoost** (1000 iterations, maximum depth of 6 trees, symmetric tree growing policy) | L2 leaf regularization = 6.0  Learning rate = 0.00001  Random Strength = 2.0 | Fold 1: 0.91975309  Fold 2: 0.79629630  Fold 3: 0.77777778  Fold 4: 0.80555556  Fold 5: 0.90123457 | **0.84 (0.73, 0.96)** |
| **Features: Mid-upper arm circumference at mid-gestation + BMI at mid-gestation + Mean arterial blood pressure at mid-gestation Diagnosis of gestational diabetes mellitus (WHO 1999) (MUAC_BMI_MAP_GDM)** | **Hyperparameters tuned using grid search** | **5-fold stratified cross validation scores of best estimator (AUC)** | **Average AUC (95% CI)** |
| **Model Specifications** |  |  |  |
| Logistic Regression (L2 regularization penalty, stochastic average gradient descent solver) | Inverse of regularization strength = 2.0 | Fold 1: 0.93827160  Fold 2: 0.83950617  Fold 3: 0.85185185  Fold 4: 0.77777778  Fold 5: 0.87037037 | 0.86 (0.76, 0.96) |
| Support Vector Machine (linear kernel, L2 regularization penalty) | L2 regularization penalty = 1.0  Loss function = ‘squared hinge’ | Fold 1: 0.93209877  Fold 2: 0.83333333  Fold 3: 0.85185185  Fold 4: 0.77777778  Fold 5: 0.86419753 | 0.85 (0.75, 0.95) |
| Neural Network (3 hidden layers with 10 neurons each, ReLU activation function, Adam solver, 200 iterations) | L2 regularization penalty = 0.001  Initial learning rate = 0.1 | Fold 1: 0.90740741  Fold 2: 0.78395062  Fold 3: 0.85185185  Fold 4: 0.76543210  Fold 5: 0.85802469 | 0.84 (0.73, 0.95) |
| **CatBoost** (1000 iterations, maximum depth of 6 trees, symmetric tree growing policy) | L2 leaf regularization = 4.0  Learning rate = 0.00001  Random Strength = 1.0 | Fold 1: 0.93209877  Fold 2: 0.86419753  Fold 3: 0.83950617  Fold 4: 0.78395062  Fold 5: 0.87037037 | **0.86 (0.76, 0.95)** |

**Supplementary Table S5: Sensitivity Analysis (AGM)**

| **Features: BMI at mid-gestation + Diagnosis of gestational diabetes mellitus (modified two-point IADPSG 2018) (BMI_GDM2)** | **Hyperparameters tuned using grid search** | **5-fold stratified cross validation scores of best estimator (AUC)** | **Average AUC (95% CI)** |
| --- | --- | --- | --- |
| **Model Specifications** |  |  |  |
| Logistic Regression (L2 regularization penalty, stochastic average gradient descent solver) | Inverse of regularization strength = 1.0 | Fold 1: 0.77159468  Fold 2: 0.73920266  Fold 3: 0.76578073  Fold 4: 0.76079734  Fold 5: 0.80564784 | 0.77 (0.73, 0.81) |
| Support Vector Machine (linear kernel, L2 regularization penalty) | L2 regularization penalty = 1.0  Loss function = ‘hinge’ | Fold 1: 0.77159468  Fold 2: 0.73920266  Fold 3: 0.76578073  Fold 4: 0.76079734  Fold 5: 0.80564784 | 0.77 (0.73, 0.81) |
| Neural Network (3 hidden layers with 10 neurons each, ReLU activation function, Adam solver, 200 iterations) | L2 regularization penalty = 0.1  Initial learning rate = 0.01 | Fold 1: 0.77325581  Fold 2: 0.73920266  Fold 3: 0.76578073  Fold 4: 0.76079734  Fold 5: 0.81063123 | 0.77 (0.72, 0.82) |
| **CatBoost** (1000 iterations, maximum depth of 6 trees, symmetric tree growing policy) | L2 leaf regularization = 2.0  Learning rate = 0.03  Random Strength = 5.0 | Fold 1: 0.76910299  Fold 2: 0.79651163  Fold 3: 0.86129568  Fold 4: 0.75996678  Fold 5: 0.76993355 | **0.79 (0.72, 0.86)** |
| **Features: BMI at mid-gestation + Fasting Glucose (BMI_Fasting)** | **Hyperparameters tuned using grid search** | **5-fold stratified cross validation scores of best estimator (AUC)** | **Average AUC (95% CI)** |
| **Model Specifications** |  |  |  |
| Logistic Regression (L2 regularization penalty, stochastic average gradient descent solver) | Inverse of regularization strength = 1.0 | Fold 1: 0.67774086  Fold 2: 0.61627907  Fold 3: 0.80564784  Fold 4: 0.84219269  Fold 5: 0.82059801 | 0.75 (0.58, 0.93) |
| Support Vector Machine (linear kernel, L2 regularization penalty) | L2 regularization penalty = 1.0  Loss function = ‘squared hinge’ | Fold 1: 0.64451827  Fold 2: 0.62624585  Fold 3: 0.78737542  Fold 4: 0.83222591  Fold 5: 0.81727575 | 0.74 (0.57, 0.92) |
| Neural Network (3 hidden layers with 10 neurons each, ReLU activation function, Adam solver, 200 iterations) | L2 regularization penalty = 0.00001  Initial learning rate = 0.01 | Fold 1: 0.64950166  Fold 2: 0.57641196  Fold 3: 0.80066445  Fold 4: 0.83720930  Fold 5: 0.82724252 | 0.75 (0.57, 0.93) |
| **CatBoost** (1000 iterations, maximum depth of 6 trees, symmetric tree growing policy) | L2 leaf regularization = 3.0  Learning rate = 0.01  Random Strength = 1.0 | Fold 1: 0.68853821  Fold 2: 0.65780731  Fold 3: 0.89617940  Fold 4: 0.90863787  Fold 5: 0.80564784 | **0.79 (0.59, 0.99)** |
| **Features: BMI at mid-gestation + Fasting Glucose (BMI_2hour)** | **Hyperparameters tuned using grid search** | **5-fold stratified cross validation scores of best estimator (AUC)** | **Average AUC (95% CI)** |
| **Model Specifications** |  |  |  |
| Logistic Regression (L2 regularization penalty, stochastic average gradient descent solver) | Inverse of regularization strength = 8.0 | Fold 1: 0.77740864  Fold 2: 0.73588040  Fold 3: 0.72093023  Fold 4: 0.74086379  Fold 5: 0.83554817 | 0.76 (0.68, 0.84) |
| Support Vector Machine (linear kernel, L2 regularization penalty) | L2 regularization penalty = 6.0  Loss function = ‘squared hinge’ | Fold 1: 0.77740864  Fold 2: 0.73588040  Fold 3: 0.72093023  Fold 4: 0.74086379  Fold 5: 0.83554817 | 0.76 (0.68, 0.84) |
| Neural Network (3 hidden layers with 10 neurons each, ReLU activation function, Adam solver, 200 iterations) | L2 regularization penalty = 0.001  Initial learning rate = 0.1 | Fold 1: 0.77740864  Fold 2: 0.73255814  Fold 3: 0.71428571  Fold 4: 0.73754153  Fold 5: 0.83056478 | 0.76 (0.68, 0.85) |
| **CatBoost** (1000 iterations, maximum depth of 6 trees, symmetric tree growing policy) | L2 leaf regularization = 6.0  Learning rate = 0.001  Random Strength = 1.0 | Fold 1: 0.78737542  Fold 2: 0.73588040  Fold 3: 0.69767442  Fold 4: 0.75415282  Fold 5: 0.81561462 | **0.76 (0.68, 0.84)** |
| **Features: BMI at mid-gestation + Fasting Glucose + 2-hour Glucose (BMI_Fasting_2hour)** | **Hyperparameters tuned using grid search** | **5-fold stratified cross validation scores of best estimator (AUC)** | **Average AUC (95% CI)** |
| **Model Specifications** |  |  |  |
| Logistic Regression (L2 regularization penalty, stochastic average gradient descent solver) | Inverse of regularization strength = 2.0 | Fold 1: 0.75415282  Fold 2: 0.75415282  Fold 3: 0.72923588  Fold 4: 0.74418605  Fold 5: 0.86544850 | 0.77 (0.67, 0.87) |
| Support Vector Machine (linear kernel, L2 regularization penalty) | L2 regularization penalty = 3.0  Loss function = ‘squared hinge’ | Fold 1: 0.73421927  Fold 2: 0.74916944  Fold 3: 0.73255814  Fold 4: 0.75249169  Fold 5: 0.86378738 | 0.77 (0.67, 0.86) |
| Neural Network (3 hidden layers with 10 neurons each, ReLU activation function, Adam solver, 200 iterations) | L2 regularization penalty = 0.01  Initial learning rate = 0.01 | Fold 1: 0.76578073  Fold 2: 0.74584718  Fold 3: 0.71594684  Fold 4: 0.74916944  Fold 5: 0.84883721 | 0.77 (0.68, 0.85) |
| **CatBoost** (1000 iterations, maximum depth of 6 trees, symmetric tree growing policy) | L2 leaf regularization = 4.0  Learning rate = 0.001  Random Strength = 5.0 | Fold 1: 0.76079734  Fold 2: 0.75249169  Fold 3: 0.75415282  Fold 4: 0.76744186  Fold 5: 0.85714286 | **0.78 (0.70, 0.86)** |
| **Features: Pre-pregnancy BMI (ppBMI)** | **Hyperparameters tuned using grid search** | **5-fold stratified cross validation scores of best estimator (AUC)** | **Average AUC (95% CI)** |
| **Model Specifications** |  |  |  |
| Logistic Regression (L2 regularization penalty, stochastic average gradient descent solver) | Inverse of regularization strength = 1.0 | Fold 1: 0.84807692  Fold 2: 0.72115385  Fold 3: 0.65096154  Fold 4: 0.71346154  Fold 5: 0.60096154 | 0.71 (0.54, 0.87) |
| Support Vector Machine (linear kernel, L2 regularization penalty) | L2 regularization penalty = 1.0  Loss function = ‘hinge’ | Fold 1: 0.84807692  Fold 2: 0.72115385  Fold 3: 0.65096154  Fold 4: 0.71346154  Fold 5: 0.60096154 | 0.71 (0.54, 0.87) |
| Neural Network (3 hidden layers with 10 neurons each, ReLU activation function, Adam solver, 200 iterations) | L2 regularization penalty = 0.0001  Initial learning rate = 0.1 | Fold 1: 0.85288462  Fold 2: 0.72115385  Fold 3: 0.65096154  Fold 4: 0.71346154  Fold 5: 0.60096154 | 0.71 (0.54, 0.87) |
| **CatBoost** (1000 iterations, maximum depth of 6 trees, symmetric tree growing policy) | L2 leaf regularization = 1.0  Learning rate = 0.05  Random Strength = 6.0 | Fold 1: 0.84423077  Fold 2: 0.75961538  Fold 3: 0.69711538  Fold 4: 0.67307692  Fold 5: 0.62788462 | **0.72 (0.57, 0.87)** |

**Supplementary Table S6: Sensitivity Analysis (T2D)**

| **Features: BMI at mid-gestation + Diagnosis of gestational diabetes mellitus (modified two-point IADPSG 2018) (BMI_GDM2)** | **Hyperparameters tuned using grid search** | **5-fold stratified cross validation scores of best estimator (AUC)** | **Average AUC (95% CI)** |
| --- | --- | --- | --- |
| **Model Specifications** |  |  |  |
| Logistic Regression (L2 regularization penalty, stochastic average gradient descent solver) | Inverse of regularization strength = 1.0 | Fold 1: 0.83950617  Fold 2: 0.88888889  Fold 3: 0.83333333  Fold 4: 0.76851852  Fold 5: 0.95679012 | 0.86 (0.73, 0.98) |
| Support Vector Machine (linear kernel, L2 regularization penalty) | L2 regularization penalty = 1.0  Loss function = ‘squared hinge’ | Fold 1: 0.83950617  Fold 2: 0.88888889  Fold 3: 0.83333333  Fold 4: 0.76851852  Fold 5: 0.95679012 | 0.86 (0.73, 0.98) |
| Neural Network (3 hidden layers with 10 neurons each, ReLU activation function, Adam solver, 200 iterations) | L2 regularization penalty = 0.1  Initial learning rate = 0.1 | Fold 1: 0.83950617  Fold 2: 0.89506173  Fold 3: 0.83333333  Fold 4: 0.76851852  Fold 5: 0.95061728 | 0.86 (0.74, 0.98) |
| **CatBoost** (1000 iterations, maximum depth of 6 trees, symmetric tree growing policy) | L2 leaf regularization = 3.0  Learning rate = 0.00001  Random Strength = 1.0 | Fold 1: 0.83950617  Fold 2: 0.84567901  Fold 3: 0.80864198  Fold 4: 0.76851852  Fold 5: 0.95679012 | **0.84 (0.72, 0.97)** |
| **Features: BMI at mid-gestation + Fasting Glucose (BMI_Fasting)** | **Hyperparameters tuned using grid search** | **5-fold stratified cross validation scores of best estimator (AUC)** | **Average AUC (95% CI)** |
| **Model Specifications** |  |  |  |
| Logistic Regression (L2 regularization penalty, stochastic average gradient descent solver) | Inverse of regularization strength = 1.0 | Fold 1: 0.74691358  Fold 2: 0.91358025  Fold 3: 0.79629630  Fold 4: 0.69753086  Fold 5: 0.62962963 | 0.76 (0.57, 0.95) |
| Support Vector Machine (linear kernel, L2 regularization penalty) | L2 regularization penalty = 1.0  Loss function = ‘squared hinge’ | Fold 1: 0.73456790  Fold 2: 0.86419753  Fold 3: 0.59259259  Fold 4: 0.67283951  Fold 5: 0.67901235 | 0.71 (0.53, 0.89) |
| Neural Network (3 hidden layers with 10 neurons each, ReLU activation function, Adam solver, 200 iterations) | L2 regularization penalty = 0.00001  Initial learning rate = 0.1 | Fold 1: 0.75925926  Fold 2: 0.88888889  Fold 3: 0.72222222  Fold 4: 0.69753086  Fold 5: 0.64814815 | 0.74 (0.58, 0.90) |
| **CatBoost** (1000 iterations, maximum depth of 6 trees, symmetric tree growing policy) | L2 leaf regularization = 4.0  Learning rate = 0.001  Random Strength = 1.0 | Fold 1: 0.75308642  Fold 2: 0.88888889  Fold 3: 0.79938272  Fold 4: 0.66666667  Fold 5: 0.70987654 | **0.76 (0.61, 0.91)** |
| **Features: BMI at mid-gestation + 2-hour Glucose (BMI_2hour)** | **Hyperparameters tuned using grid search** | **5-fold stratified cross validation scores of best estimator (AUC)** | **Average AUC (95% CI)** |
| **Model Specifications** |  |  |  |
| Logistic Regression (L2 regularization penalty, stochastic average gradient descent solver) | Inverse of regularization strength = 10.0 | Fold 1: 0.93209877  Fold 2: 0.91358025  Fold 3: 0.80246914  Fold 4: 0.81481481  Fold 5: 0.88271605 | 0.87 (0.77, 0.97) |
| Support Vector Machine (linear kernel, L2 regularization penalty) | L2 regularization penalty = 3.0  Loss function = ‘hinge’ | Fold 1: 0.90123457  Fold 2: 0.92592593  Fold 3: 0.82098765  Fold 4: 0.82098765  Fold 5: 0.85802469 | 0.87 (0.78, 0.95) |
| Neural Network (3 hidden layers with 10 neurons each, ReLU activation function, Adam solver, 200 iterations) | L2 regularization penalty = 0.001  Initial learning rate = 0.01 | Fold 1: 0.93209877  Fold 2: 0.90740741  Fold 3: 0.80246914  Fold 4: 0.81481481  Fold 5: 0.88271605 | 0.87 (0.77, 0.97) |
| **CatBoost** (1000 iterations, maximum depth of 6 trees, symmetric tree growing policy) | L2 leaf regularization = 6.0  Learning rate = 0.0001  Random Strength = 6.0 | Fold 1: 0.91358025  Fold 2: 0.89814815  Fold 3: 0.82098765  Fold 4: 0.77777778  Fold 5: 0.87654321 | **0.86 (0.76, 0.96)** |
| **Features: BMI at mid-gestation + Fasting Glucose + 2-hour Glucose (BMI_Fasting_2hour)** | **Hyperparameters tuned using grid search** | **5-fold stratified cross validation scores of best estimator (AUC)** | **Average AUC (95% CI)** |
| **Model Specifications** |  |  |  |
| Logistic Regression (L2 regularization penalty, stochastic average gradient descent solver) | Inverse of regularization strength = 3.0 | Fold 1: 0.93209877  Fold 2: 0.88888889  Fold 3: 0.70987654  Fold 4: 0.79629630  Fold 5: 0.85802469 | 0.84 (0.69, 0.99) |
| Support Vector Machine (linear kernel, L2 regularization penalty) | L2 regularization penalty = 2.0  Loss function = ‘squared hinge’ | Fold 1: 0.93209877  Fold 2: 0.87037037  Fold 3: 0.66049383  Fold 4: 0.77777778  Fold 5: 0.82098765 | 0.81 (0.63, 0.99) |
| Neural Network (3 hidden layers with 10 neurons each, ReLU activation function, Adam solver, 200 iterations) | L2 regularization penalty = 0.00001  Initial learning rate = 0.01 | Fold 1: 0.92592593  Fold 2: 0.82098765  Fold 3: 0.58024691  Fold 4: 0.80864198  Fold 5: 0.81481481 | 0.84 (0.69, 0.99) |
| **CatBoost** (1000 iterations, maximum depth of 6 trees, symmetric tree growing policy) | L2 leaf regularization = 5.0  Learning rate = 0.0001  Random Strength = 5.0 | Fold 1: 0.90123457  Fold 2: 0.87037037  Fold 3: 0.80864198  Fold 4: 0.75925926  Fold 5: 0.85185185 | **0.84 (0.74, 0.94)** |
| **Features: Pre-pregnancy BMI (ppBMI)** | **Hyperparameters tuned using grid search** | **5-fold stratified cross validation scores of best estimator (AUC)** | **Average AUC (95% CI)** |
| **Model Specifications** |  |  |  |
| Logistic Regression (L2 regularization penalty, stochastic average gradient descent solver) | Inverse of regularization strength = 1.0 | Fold 1: 0.53333333  Fold 2: 0.65333333  Fold 3: 0.73333333  Fold 4: 0.48000000  Fold 5: 0.74000000 | 0.63 (0.42, 0.83) |
| Support Vector Machine (linear kernel, L2 regularization penalty) | L2 regularization penalty = 1.0  Loss function = ‘hinge’ | Fold 1: 0.53333333  Fold 2: 0.65333333  Fold 3: 0.73333333  Fold 4: 0.48000000  Fold 5: 0.74000000 | 0.63 (0.42, 0.83) |
| Neural Network (3 hidden layers with 10 neurons each, ReLU activation function, Adam solver, 200 iterations) | L2 regularization penalty = 0.00001  Initial learning rate = 0.1 | Fold 1: 0.53333333  Fold 2: 0.62333333  Fold 3: 0.73333333  Fold 4: 0.48000000  Fold 5: 0.74000000 | 0.63 (0.42, 0.83) |
| **CatBoost** (1000 iterations, maximum depth of 6 trees, symmetric tree growing policy) | L2 leaf regularization = 1.0  Learning rate = 0.00001  Random Strength = 3.0 | Fold 1: 0.55333333  Fold 2: 0.62666667  Fold 3: 0.75333333  Fold 4: 0.43333333  Fold 5: 0.75000000 | **0.62 (0.39, 0.86)** |
